# Patients’ worries before starting antiretroviral therapy and their association with treatment adherence and outcomes: a prospective study in rural Uganda, 2004 - 2009

**DOI:** 10.1186/1756-0500-6-187

**Published:** 2013-05-07

**Authors:** Billy N Mayanja, Kenneth Ekoru, Harriet Namugenyi, Rosemary Lubega, Joseph O Mugisha

**Affiliations:** 1 MRC/UVRI Uganda Research Unit on AIDS, P.O. Box 49, Entebbe, Uganda

**Keywords:** Antiretroviral therapy, Patients’ worries, Adherence, Treatment outcomes

## Abstract

**Background:**

In HIV-infected persons, good adherence to antiretroviral therapy (ART) is essential for successful treatment outcomes. Patients’ worries before starting ART may affect their ART adherence and treatment outcomes.

**Methods:**

Between 2004 and 2009, HIV-infected individuals in a prospective cohort study in rural Uganda were assessed for ART eligibility. A counsellor explained the ART eligibility criteria, adherence and side effects, and recorded the patients’ worries related to ART. Every quarter, patients who initiated ART had clinical, immunological (CD4 cell counts) and virological (viral loads) assessments, and data were collected on ART adherence using patients’ self-reports and pill counts. We describe the patients’ worries and examine their association with ART adherence, and immunological and virological outcomes.

**Results:**

We assessed 421 patients, 271 (64%) were females, 318 (76%) were aged 30 years and above and 315 (75%) were eligible for ART. 277 (66%) reported any worry, and the proportions were similar by sex, age group and ART eligibility status. The baseline median CD4 counts and viral loads were similar among patients with any worry and those with no worry. The commonest worries were: fear of HIV serostatus disclosure; among 69 (16%) participants, lack of food when appetite improved after starting ART; 50 (12%), concurrent use of other medications; 33 (8%), adherence to ART; 28 (7%) and problems concerning condom use; 27 (6%). After 24 months or more on ART, patients who reported any worry had made more scheduled ART refill visits than patients who reported no worry (p<0.01), but the annual CD4 cell increases were similar (p=0.12). After one year on ART, patients who reported any worry had greater virological suppression than patients who reported no worry (p<0.05).

**Conclusions:**

Despite the lack of significant associations of worries with unfavourable ART outcomes, physicians and counsellors should assist patients in overcoming their worries that can cause stress and discomfort. Food supplements may be desirable for some patients initiating ART.

## Background

In HIV-infected persons, good adherence to antiretroviral therapy (ART) is essential for successful treatment outcomes and improved quality of life with reduced morbidity and mortality [1-3]. Poor ART adherence is associated with perceived HIV stigma among patients receiving ART [4], being single (never married) and belief in divine spiritual healing in Uganda [5,6], and in Cote d’Ivoire, fear of treatment side effects and use of traditional healers [7]. Good ART adherence was associated with formal education level in Nigeria [8] and concerns for family well being in Uganda [9]. Common barriers to ART adherence in developed and developing countries were found to be; fear of disclosure, forgetfulness, lack of understanding of treatment benefits and leaving behind medications, while problems of access, including financial constraints and disruption in drug supplies were specific to developing countries [10].

There are no published reports that have described patients’ worries before starting ART and how they affect ART adherence and treatment outcomes, because most studies on ART adherence have been among patients already on ART. However, prior to starting ART, it is important to assess patients’ beliefs and knowledge about ART to prepare them for the life-long treatment and to enhance adherence [11,12]. We describe the worries reported by adult patients at the first assessment for ART eligibility and also examine the possible association of these worries with; ART adherence, and immunological and virological ART outcomes. This information will be useful in the counselling and assessment of patients for the lifelong ART.

## Methods

In January 2004, free ART was introduced for eligible participants in a prospective HIV-1 clinical cohort and general population cohort in rural Uganda [13-15]. Participants’ assessment for ART eligibility involved ART counselling on: eligibility criteria, life-long treatment, effects and side-effects, adherence, safe sex practices, nutrition, contraception, HIV serostatus disclosure, need for treatment supporter and couple counselling. At the assessment for ART eligibility, participants’ questions, concerns, worries or comments related to ART were recorded on a standardized check-list form. World Health Organisation (WHO) HIV clinical staging [16] and CD4 cell count measurements were done, and cotrimoxazole prophylaxis was provided [17]. Participants who were not yet eligible to start ART were given a date for future reassessment while eligible participants initiated ART according to the Uganda National ART guidelines [18,19]. Participants were reviewed quarterly for clinical and laboratory assessment, monthly for ART refills and whenever they fell sick. We defined patient’s worries before starting ART as the patient’s reported questions, worries, concerns and comments regarding ART during the first ART eligibility assessment.

### Categorisation of participants’ worries

Although participants asked open questions or expressed their worries or concerns openly, these were categorised as worries related to: (a) Fear of HIV serostatus disclosure by the patient. (b) Food and nutrition; worry over lack of food when appetite improved while receiving ART. (c) Condom use; concerns or worries about using condoms; either avoiding disclosing HIV serostatus, not knowing how to use them, or reluctance to use them. (d) Concurrent use of other medications like cotrimoxazole prophylaxis, anti-tuberculosis drugs, anti-hypertensive drugs, herbal medications etc. (e) Worry about ART adherence; especially taking the ART medications promptly and for the rest of their lives. (f) Worry of getting ARV side effects. (g) Whether ARVs will actually improve their health (regain strength and weight). (h) Failure to identify a treatment supporter. (i) Contraception /child bearing; concerns whether one would improve and be able bear a child, and what interval one can take to conceive after starting ART.

### Measurement of outcomes

The primary outcome measure was ART adherence while the secondary outcome measures were the immunological (CD4 cell counts), virological (viral loads) treatment outcomes, ART side effects and death. At each ART refill visit, we collected ART adherence data on: whether the participant was late for the ART refill visit, the number of days the participant missed taking pills, reasons for missing pills and did a pill count. Data was collected on any side effects related to ART and also on deaths that occurred after starting ART. A full blood count was done at ART initiation, one month and quarterly. CD4 cell counts were measured at ART initiation and quarterly using the FACS Count method (Becton Dickinson, San Jose, CA, USA), with external quality control from the United Kingdom External Quality Assurance Scheme (UKNEQAS). Viral loads were measured at ART initiation and every 6 months. From January 2004 to September 2007, we used the VERSANT RNA 3.0 (Bayer, Bayer HealthCare, NY, USA) assay (lower detection limit of 50 copies/ml). While from October 2007, we used the COBAS Amplicor MONITOR 1.5 (Roche, Roche Molecular Systems, NJ, USA) assay (lower detection limit of 400 copies/ml). External quality assurance was from the Virology Quality Assurance Scheme (Rush University, Chicago, IL) and UKNEQAS.

### Statistical analysis

Data was managed using a Microsoft Access data base and analysed using Stata version 11. Baseline characteristics, WHO HIV clinical stage, CD4 cell counts and log viral loads of participants who reported ART worries and those who reported no worries were compared. Overall and specific worries were examined by ART eligibility status and gender. We used two approaches to compare ART adherence among those with no worry and those with one worry or more; (a) we compared the proportion of patients who made a given number of scheduled ART refill visits and (b) also the proportions with any self-reported lateness for appointment at least once, having missed ART doses in 4 days prior to the visit and having missed ART doses in the weekend prior to the visit. Mean CD4 increase and median log viral load decrease were compared between patients with worries and those with no worry, and between patients with a specific worry and all those without that specific worry. Proportions were compared using the Chi squared tests except where a specific worry was reported by fewer than 5 patients when Fisher’s Exact test was used. We assessed the association between patients’ worries and; ART adherence, immunological outcomes, virological outcomes, ART side-effects and death, adjusting for potential confounders; age, sex, WHO HIV clinical stage and CD4 cell counts.

### Ethical consideration

The study was approved by the Science and Ethics committee of the Uganda Virus Research Institute and by the Uganda National Council for Science and Technology. Participants gave informed written consent for participation in the study and confidentiality was ensured throughout the study.

## Results

Between January 2004 and October 2009, we assessed 421 HIV-infected individuals for ART eligibility: 271 (64%) were female, 318 (76%) were aged 30 years and older (Table 1). The age distribution of individuals who were eligible to start ART at the first assessment for ART eligibility and those who were not eligible was similar (Table 1). There were no significant differences in the distribution of ART eligibility status, patients’ gender, median CD4 cell counts and median viral loads between individuals who had no worries and those who had one worry or more. Of 421 people assessed for ART eligibility, 277 (66%) expressed one worry or more about taking ART. The commonest worries reported were: fear of HIV serostatus disclosure in 69 (16%) patients, lack of food when appetite improved in 50 (12%), concurrent use of other medications in 33 (8%), ART adherence in 28 (7%), condom use (avoiding disclosing HIV serostatus, not knowing how to use them, or reluctance to use them) in 27 (6%), and antiretroviral (ARV) drug side-effects in 23 (5%) (Table 2).

**Table 1:** Patients’ characteristics at first assessment for ART eligibility

	**ART eligibility status**					
**Patients’ characteristics**	**Eligible**		**Not eligible**		**Total**	
	**n**	**(%)**	**n**	**(%)**	**n**	**(%)**
Total	217	(52)	204	(48)	421	(100)
Gender						
Males	79	(36)	71	(35)	150	(36)
Females	138	(64)	133	(65)	271	(64)
WHO clinical stage						
1	38	(18)	109	(53)	147	(35)
2	64	(29)	67	(33)	131	(31)
3	81	(37)	28	(14)	109	(26)
4	34	(16)	0	(0)	34	(8)
Age (years)						
13-19	7	(3)	3	(1)	10	(2)
20-29	53	(24)	40	(20)	93	(22)
30-39	81	(37)	90	(44)	171	(22)
40 +	76	(35)	71	(35)	147	(35)
Mean (SD)	37.0 (11.8)		37.0 (10.4)		37.0 (11.1)	
Median [IQR]	35.0 [29-43]		36.0 [30-43]		35.0 [30-43]	
Range	14 – 78		14 – 72		14-78	
CD4 cell counts/μL^1^						
<200	186	(86)	0	(0)	186	(45)
≥200	30	(14)	202	(100)	232	(56)
Mean (SD)	127 (155)		519 (254)		317 (287)	
Median [IQR]	114 [37-179]		466 [314-663]		225 [107-466]	
Range	1 – 1764		201 – 1298		1-1764	

*SD* - standard deviation *IQR* - inter-quartile range.

^1^One person eligible for ART had CD4 missing while 2 people not eligible for ART had CD4 missing.

Some percentages do not add up to exactly 100 because of rounding off errors.

**Table 2:** Distribution of patients’ worries at the first assessment for ART eligibility; by eligibility status and gender

**Patient’s reported worries**	**Total**		**ART eligibility status**					**Gender**				
			**Eligible**		**Not eligible**		**p-value**	**Males**		**Females**		**p-value**
	**n**	**(%)**	**n**	**(%)**	**n**	**(%)**		**n**	**(%)**	**n**	**(%)**	
Total	421		217		204			150		271		
Any worry	277	(66)	141	(65)	136	(67)	0.715	95	(63)	182	(67)	0.428
HIV serostatus disclosure	69	(16)	38	(18)	31	(15)	0.521	23	(15)	46	(17)	0.663
Food and nutrition	50	(12)	25	(12)	25	(12)	0.816	14	(9)	36	(13)	0.230
Use of other medications	33	(8)	20	(9)	13	(6)	0.278	14	(9)	19	(7)	0.396
ART adherence	28	(7)	14	(6)	14	(7)	0.886	8	(5)	20	(7)	0.420
Condom use	27	(6)	13	(6)	14	(7)	0.715	11	(7)	16	(6)	0.566
ARV side effects	23	(5)	15	(7)	8	(4)	0.177	4	(3)	19	(7)	0.600
Will ARVs make me better?	20	(5)	13	(6)	7	(3)	0.217	6	(4)	14	(5)	0.590
Treatment supporter	13	(3)	4	(2)	9	(4)	0.128	5	(3)	8	(3)	0.829
Contraception /child bearing	14	(3)	6	(3)	8	(4)	0.508	3	(2)	11	(4)	0.259
Will ARVS make me worse?	13	(3)	5	(2)	8	(4)	0.338	4	(3)	9	(3)	0.710
Residence outside study area	12	(3)	4	(2)	8	(4)	0.200	1	(1)	11	(4)	0.063*
Alcohol use	11	(3)	4	(2)	7	(3)	0.307	7	(5)	4	(1)	0.049
Why not start ART immediately	11	(3)	4	(2)	7	(3)	0.307	4	(3)	7	(3)	0.959
Employment/work issues	6	(1)	4	(2)	2	(1)	0.455	4	(3)	2	(1)	0.110
Spouse’s HIV serostatus	5	(1)	3	(1)	2	(1)	0.990*	2	(1)	3	(1)	0.990 *
Pill burden	3	(1)	3	(1)	0	(0)	0.249*	1	(1)	2	(1)	0.990*
HIV-related stigma	3	(1)	2	(1)	1	(0)	0.599	0		3	(1)	0.196
Cigarette smoking	1	(0)	0	(0)	1	(0)	0.485*	1	(1)	0		0.356 *
Others	50	(12)	23	(11)	27	(13)	0.403	15	(10)	35	(13)	0.376

* Fisher’s exact test.

Note: numbers do not add up as some patients had more than one worry.

**Figure 1:**
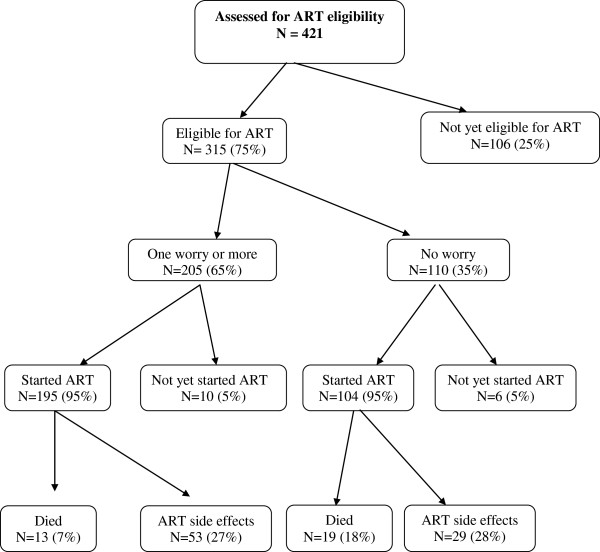
Flow chart of study participants.

Of the 421 patients assessed for ART eligibility, 315 (75%) were found to be eligible for ART (217 at the first assessment and 98 at a subsequent one). By October 2009, of the 315 patients found to be eligible for ART, 299 (95%) [104 (35%) with no worry and 195 (65%) with one or more worries] started ART had started ART (Figure 1) and 225 (71%) had completed 12 months of follow-up while 172 (55%) had completed at least 24 months of follow-up. Among the 225 patients who had completed 12 months on ART, there was no association between ART adherence and prior worries about ART (Table 3). However in the 172 patients who had completed 24 months on ART, a higher proportion 22% (26/120) of those who had at least one worry or more had attended 23 or more scheduled visits compared to 6% (3/52) among those without any worry (p<0.01). There was no association between the self-reported ART adherence measures and having any specific worry among patients who had completed either 12 or 24 months on ART.

**Table 3:** ART adherence in patients with and without worries, and those with selected specific worries stratified by duration on ART

**ART adherence**	**Worries reported at first assessment for ART eligibility**															
	**No worry**		**One worry or more**		**One worry or more**											
					**HIV status disclosure**		**Nutrition**		**Condoms use**		**Use of other medication**		**ART adherence**		**ART side effects**	
	**n=110**		**n=205**		**n=54**		**n=38**		**n=23**		**n=26**		**n=19**		**n=19**	
No of participants:	n	(%)	n	(%)	n	(%)	n	(%)	n	(%)	n	(%)	n	(%)	n	(%)
Eligible but not yet started ART	6	(5)	10	(5)	3	(6)	0	(0)	0	(0)	2	(8)	0	(0)	0	(0)
Started < 12 months ago	28	(25)	46	(22)	12	(22)	9	(24)	4	(17)	6	(23)	2	(11)	7	(37)
Started 12-24 months	24	(22)	29	(14)	7	(13)	7	(18)	6	(26)	3	(12)	3	(16)	1	(5)
Completed 24 months	52	(47)	120	(59)	32	(59)	22	(58)	13	(57)	15	(58)	14	(74)	11	(58)
**Patients who had completed 12 months on ART**	**76**		**149**		**39**		**29**		**19**		**18**		**17**		**12**	
No of scheduled visits in first year																
<9 visits	31	(41)	55	(37)	14	(36)	6	(21)	9	(47)	6	(33)	6	(35)	5	(42)
9-10 visits	20	(26)	45	(30)	12	(31)	12	(41)	3	(16)	3	(17)	7	(41)	5	(42)
11 or more	25	(33)	49	(33)	13	(33)	11	(38)	7	(37)	9	(50)	4	(24)	2	(17)
Self-reported ART adherence																
Late for appointment at least once^a^	0		0		0		0		0		0		0		0	
Missed doses in 4 days prior to visit	18	(24)	27	(18)	8	(21)	3	(10)	4	(21)	5	(28)	3	(18)	3	(25)
Missed doses in weekend prior to visit	0	0		0		0		0		0			0	0		
**Patients who had completed 24 months on ART**	**52**		**120**		**32**		**22**		**13**		**15**		**14**		**11**	
No of scheduled visits #		*														
<20	22	(42)	60	(50)	17	(53)	9	(41)	6	(46)	6	(40)	5	(36)	7	(64)
20-22	27	(52)	34	(28)	7	(22)	9	(41)	4	(31)	3	(20)	6	(43)	3	(27)
≥23	3	(6)	26	(22)	8	(25)	4	(18)	3	(23)	6	(40)	3	(21)	1	(9)
Self-reported ART adherence																
Late for appointment at least once	0		0		0		0		0		0		0		0	
Missed doses in 4 days prior to visit	19	(37)	38	(32)	14	(44)	6	(27%)	4	(31)	3	(20)	4	(29)	3	(27)
Missed doses in weekend prior to visit	0		0		0		0		0		0		0		0	

Note: comparisons are between those with one or more worries and those with no worry, and also between those with a particular worry and all those without that particular worry.

^a^ 4 missing data for all adherence data (late appointments, missed doses) in the first year.

* p<0.01.

# Comparisons using chi-squared test on 2 degrees of freedom, comparing the percentage in each of the three categories.

Of the 299 patients who started ART (104 with no worry and 195 with one or more worries), CD4 cell counts at 1 year after ART initiation were available for 197 (66%), (63 with no worry and 134 with one or more worries). There was no association between mean CD4 counts increases per year and reporting any worry at the first assessment for ART eligibility (Table 4). Viral load data after 1 year on ART was available for 180 patients (54 with no worry and 126 with one worry or more). Individuals who reported one worry or more was associated with greater reductions in log viral load, p<0.05. However this association was more marked among individuals who were worried about HIV serostatus disclosure and nutrition who had a greater reduction compared to those without any of these worries p<0.05 for both). ART regimen change due to side effects or treatment failure (substitution or switch) and death were not associated with reporting a worry (Table 4).

**Table 4:** Comparison of ART outcomes after 1 year between those with one or more worries and those without any worry at the first eligibility assessment

**ART outcomes**	**Worries reported at assessment for ART eligibility**							
	**No worry**	**One worry or more**	**Specific worries**					
			**HIV status disclosure**	**Nutrition**	**Condoms use**	**Use of other medication**	**ART Adherence**	**ART side effects**
	**n=104**	**n=195**	**n=54**	**n=38**	**n=23**	**n=26**	**n=19**	**n=19**
**Total with CD4 counts after ART eligibility**	**63**	**134**	**32**	**22**	**14**	**18**	**15**	**11**
Mean CD4 counts increase (per year) (95% CI)	162	199	112	127	97	363	189	156
	(112-211)	(129-270)	(80-145)	(87-167)	(42-151)	(-19-746)	(56-322)	(-31-343)
Median CD4 increase	92	114	73	112	74	122	115	168
(IQR)	(47-212)	(41-213)	(41-183)	(46-215)	(40-106)	(59-210)	(33-232)	(73-237)
**Total with viral loads**	**54**	**126**	**33**	**24**	**14**	**16**	**15**	**12**
CD4 count > 100 after ART start	42 (78%)	95 (75%)	23 (70%)	16 (67%)	10 (71%)	10 (63%)	15 (100%)	12 (100%)
Annual decrease in median log VL (IQR)	0.01	−0.10*	−0.24*	0.04*	0.18	0.17	−0.14	0.05
	(-0.10-0.12)	(-0.19 - -0.02)	(-0.41 - -0.07)	(-0.12 - -0.21)	(0.01-0.35)	(0.12 - 0.33)	(-0.49 - 0.20)	(-0.02 - 0.30)
**Number who started ART**	**104**	**195**	**51**	**38**	**23**	**24**	**19**	**19**
Substituted / switched ART	29 (28%)	53 (27%)	16 (31%)	7 (18%)	6 (26%)	8 (33%)	3 (16%)	7 (37%)
Died	19 (18%)	13 (7%)	5 (10%)	4 (11%)	1 (4%)	1 (4%)	0 (0)	1 (5%)

*p<0.05 Note: Comparisons are between those with one or more worries and those with no worry, and also between those with a particular worry and all those without that particular worry.

## Discussion

Among participants assessed for ART eligibility in this rural population based prospective cohort, small numbers reported individual worries, although about two thirds had one worry or more. The commonest worries were about fear of HIV serostatus disclosure, lack of food when appetite improved on treatment, concurrent use of other medications, ART adherence, problems concerning condom use (either avoiding disclosing HIV serostatus, not knowing how to use them, or reluctance to use them) and ARV drug side effects. Patients who had one or more worries (particularly those who were worried of HIV serostatus disclosure and lack of food when appetite improved) had greater viral load reductions than those with no worry. Among patients who had completed 24 months on ART, patients who reported one or more worries had made more scheduled ART refill visits than those with no worry, but we found no association between annual CD4 cell increases and reporting worries.

A strength of this study is that we document patients’ worries before starting ART, and the prospective study design allowed us to relate these worries to ART adherence and treatment outcomes. Also being a rural setting, our participant retention was high (over 92%), thus we had minimal loss to follow up which was not different between individuals who reported one worry or more and those who reported no worry. However, our study was limited by the small numbers of participants, and since the data were collected from a single research site, our findings may not be generalisable to other settings. Another weakness is that we did not follow up or assess to ascertain if an individual who reported a worry at the assessment for ART eligibility eventually had problems related to that worry after starting on ART. The two methods we used to assess ART adherence (pill count and patients’ self-report) as outcome measures have advantages and disadvantages; and neither is entirely reliable in determining a patient’s level of ART adherence [20,21].

A Pubmed literature search did not reveal any studies describing patients’ worries before initiating ART and their association with treatment outcomes as most studies reported findings from patients who had already started ART. It is therefore difficult to conclusively compare our findings with those from studies done in patients who had already started on ART. In our study, we did not find any association between fear of HIV serostatus disclosure and ART adherence, but studies among people already on ART have reported an association between HIV serostatus disclosure and better ART adherence [22,23]. Positive HIV serostatus disclosure to one’s spouse or sexual partner is important in preventing HIV transmission, may reduce patient’s stress, improves psychological health and leads to informed reproductive choices in couples where one or both are HIV positive [24]. We did not follow up the patients who reported worrying about HIV serostatus disclosure to ascertain whether they later disclosed to their partners or not. However, high HIV serostatus disclosure rates have been reported in studies in Uganda ( 69%) [25] and Ethiopia (94.5%), but these studies were also among individuals already on ART [26].

In our study, the proportion of patients who reported worries about lack of food after an improvement in appetite after starting ART (12%) was lower than the proportion (76%) reported in a study in Rwanda [27], however the Rwanda study was among individuals who had already initiated ART and their appetite possibly had already started improving thus leading to more of their patients being worried about lack of food.

Eight percent of our patients reported worry about concurrent use of other medications. Although we did not specifically ask what medications they were worried about using concurrently with ARV drugs, some of them may have been taking cotrimoxazole prophylaxis and/or herbal or traditional medicines of unknown therapeutic efficacy and interactions [28]. In Uganda, use of traditional herbal medications is common and in one study 33.7% of patients on ART were also reported to be using herbal medications for HIV associated symptoms [29]. Although we found no association between concurrent use of herbal treatment and ART adherence, other studies found that use of herbal medications was associated with ART non-adherence, though their use significantly declined after six months on ART [30-32]. Although only 6% of our participants reported worries about using a condom, there is need to assess actual condom use in our study population. However, our findings are collaborated by findings from another study in Uganda that reported a 70% reduction in risky sexual behaviour (defined as inconsistent or no condom use with HIV-negative or unknown HIV serostatus partners in the past 3 months) after 6 months on ART [33].

Participants may not be fully aware of the effects and side effects of ART before they start on treatment, but 7% of our participants reported worries about adherence to life-long ART. Since ART adherence is the main determinant of treatment outcomes, our failure to find any significant association between the mean annual CD4 count increases or viral load decreases and reporting worries may be due to a true lack of an association between ART adherence and reported worries. However, our finding that those participants who reported worries had greater viral load suppression than those who did not report any worry should be interpreted with caution due to the small numbers. We found that at baseline patients who reported one worry or more were less likely than those with no worries to be in WHO clinical stages 3 or 4, i.e. to have a more advanced stage of immunosuppression at ART eligibility assessment. This would mean that patients reporting worries might be more aware of, and concerned about, their deteriorating health status and thus likely to present for ART eligibility assessment. We found no association between deaths in patients on ART and reported worries.

Since ART adherence is the main determinant of treatment outcomes, our failure to find any significant differences either in the mean annual CD4 count increases or viral load decreases between those who reported one worry or more may be due to the lack of a significant association between adherence to ART and reported worries. Although we found that patients who reported one or more worries, and particularly those who were worried about HIV serostatus disclosure and nutrition had greater viral load suppression than those who did not report any worry or these two particular worries, these findings should be interpreted with caution due to the small numbers.

## Conclusions

In spite of the lack of significant associations of worries with unfavourable treatment outcomes, we detected a number of anxieties that can cause stress and discomfort to patients. Before and during ART initiation, physicians and counsellors should be aware of the worries that patients may have and assist patients in addressing them. Adoption of routine couple counselling would help overcome the worry of HIV serostatus disclosure to sexual partners and would help identify HIV discordance. Food supplements may be necessary for some patients initiating ART. Longer-term assessment is needed to evaluate the potential impact of addressing patients’ worries on the eventual adherence and treatment outcomes of the life-long ART. There is also need to follow-up patients who reported a specific worry at the time of assessment of ART eligibility to ascertain if later it became a reality after starting ART and also to examine whether there were any differences in the specific causes of death between those who had no worries and those who had one worry or more.

## Competing interests

The authors declare that they have no competing interests.

## Authors’ contributions

The authors BNM, KE, HN, RL and JOM have equally made substantial contributions to this work; in study conception and design, data acquisition, analysis and interpretation of results and manuscript preparation and revision. All authors have read and approved the final draft of the manuscript.
